# Standardizing the categorizations of models of aftercare for survivors of childhood cancer

**DOI:** 10.1186/s12913-019-4719-4

**Published:** 2019-11-20

**Authors:** Devonne Ryan, Paul C Moorehead, Roger Chafe

**Affiliations:** 1 Division of Clinical Epidemiology, Faculty of Medicine, Memorial University of Newfoundland, St. John’s, Canada; 2 Division of Pediatrics, Faculty of Medicine, Memorial University of Newfoundland, St. John’s, Canada; 3 Children’s and Women’s Health, Eastern Health, St. John’s, Canada; 4 Janeway Pediatric Research Unit, St. John’s, Canada

**Keywords:** Categorization, Childhood cancer survivor, Models of care, Aftercare, Transition

## Abstract

**Background:**

With significant improvements in the survival rates for most childhood cancers, there is increased pressure to determine how follow-up or aftercare for survivors is best structured.

**Main body:**

Previous work in this area has not been consistent in how it categorizes models of aftercare, which risks confusion between studies and evaluations of different models. The adoption of a standardized method for classifying and describing different models of aftercare is necessary in order to maximize the applicability of the available evidence. We identify some of the different ways models of aftercare have been classified in previous research. We then propose a revised taxonomy which allows for a more consistent classification and description of these models. The proposed model bases the classification of models of aftercare on who is the lead provider, and then collects data on five other key features: which other providers are involved in providing aftercare, where care is provided, how are survivors engaged, which services are provided, and who receives aftercare.

**Conclusion:**

There is a good deal of interest in the effectiveness of different models of aftercare. Future research in this area would be assisted by the adoption of a shared taxonomy that will allow programs to be identified by their structural type.

## Background

With the significant rise in the number of survivors of childhood cancer, increased attention is being given to how to structure the aftercare for these patients who face lifelong health risks [1–3]. Following the treatment of their active cancer, aftercare is initially provided by their pediatric oncology care team. In early adulthood, many survivors transition out of pediatric care, with subsequent aftercare being structured in significantly different ways across cancer programs [4]. One focus of research in this area has been on the effectiveness of different models of post-transition aftercare in supporting and serving survivors of childhood cancer [5, 6]. Yet in studying these different models of aftercare, conflicting categorizations have been employed, often without much consideration seeming to be given for the adoption of different basis for distinguishing models of aftercare. In this article, we will review the different ways models of aftercare have been categorized across various studies, and then propose a new categorization which allows for a more specific and standardized identification of program types. Finally, we discuss the possibility of employing a similar method of categorization for studying models of post-pediatric care for other chronic condition.

## Main body

### Previous Categorizations

A model of care describes the structure and type of services provided to patients with a particular condition during a period of time or phase of their disease. It broadly “defines the way health services are delivered” for a group of patients [7, 8]. Given the multiple factors that are incorporated in any model of care, it is clear that different aspects can be used to distinguish models from each other. In studying the health services available for survivors of childhood cancer, authors have previously used various aspects to distinguish models of aftercare (Table 1). One approach identifies models that are commonly employed. For example, the Institute of Medicine’s *From Cancer Patient to Cancer Survivor: Lost in Transition* took a comprehensive look at health care issues for both survivors of adult and childhood cancers [8]. In discussing models of aftercare, the authors of the report focus on “promising models of follow-up care,” and examines a shared-care model, a nurse-led model, and survivorship follow-up clinics. Similarly, in their survey of pediatric oncology centres, Eshelman-Kent et al. employ a list of models “identified in the literature [4].” Specifically, they propose using the following categories: Cancer Center-Based Model Without Community Referral, Community Referral Model, Hybrid model (Combined Cancer Center and Community Based Model), Postal/Internet/phone-based model, Adult oncologist, and Other. In their Delphi survey of policy experts, Mertens et al. used the categories chronic disease model, primary care model and late effects model [9].

**Table 1:** Previous Categorizations of Models of Care for Survivors of Childhood Cancer

Author	Basis of Categorization Identified by the Author	Models of Care Identified
Hewitt, Greenfield, Stovall [8]	“Promising models of follow-up care,”	-Shared-care model -Nurse-led model -Survivorship follow-up clinics
Eshelman-Kent et al. [4]	“Models identified in the literature”	-Cancer center-based model without community referral -Community referral model, hybrid model (combined cancer center and community-based model) -Postal/internet/phone-based model -Adult oncologist
Mertens et al. [9]	Not identified	-Chronic disease model -Primary care model -Late effects model
Oeffinger and McCabe [6]	Setting of Care	-Hospital-based model -Community-based model -Shared care model
Hahn and Ganz [10]	Setting of Care	-Academic medical center -Community hospital model -Primary-care medical group -County hospital
Wallace et al. / Michel et al. [2, 11]	Lead Provider	-Medically supervised late effects clinic -Primary care physician-led model -Nurse-led model -Postal or telephone follow-up model
Heir et al. [5]	Communication modality / Lead Provider / Setting	-Face-to-face clinic visits -Telephone, postal, email or SMS/text-based model -Physician versus nurse-led follow-up -Hospital versus primary care follow-up
Hewitt, Weiner and Simone [12]	Identified in the literature	-Comprehensive survivorship program
Aziz et al. [13]	Identified in the literature	-Comprehensive survivorship program

An alternative approach has been to identify models of care in terms of the setting in which the care is given. Oeffinger and McCabe have previously taken this approach, evaluating models of care in terms of whether they are hospital-based, community-based, or shared care, i.e., include both community and hospital care [6]. In examining the use of care plans for both survivors of childhood and adult cancer, Hahn and Ganz also distinguish models of aftercare in terms of setting: an academic medical center, a community hospital, a primary-care medical group and a county hospital [10]. Wallace et al. and Michel et al. distinguish models in terms of the profession of the person who is the lead for organizing care, e.g., medically supervised late effects clinic, primary care physician-led or nurse-led, and postal or telephone follow-up, with the appropriate level of care being dependent on the risks associated with the survivor’s type of cancer and treatment received [2, 11]. Heir et al. use a blend of different aspects to distinguish programs, including in terms of communication modalities (e.g., face-to-face clinic visits, telephone, postal, email or SMS/text-based); physician versus nurse-led follow-up; and hospital versus primary care follow-up [5].

In their 2003 Institute of Medicine report, Weiner, Simone, and Hewitt offer another approach to categorizing a model of aftercare [12]. They identify the “comprehensive survivorship program” model. Comprehensive programs are those that have “a dedicated time and place for the clinic, met at least twice a month, were staffed by a doctor with experience in the late effects after treatment for childhood cancer, had a nurse coordinator, provided state-of-the-art screening for individual’s risk of late effects, provided referrals to appropriate specialists, and provided wellness education.” Hewitt, Weiner and Simone also discuss this type of program [12]. Similarly, Aziz et al. has found this model of aftercare is commonly employed by larger pediatric oncology programs in North America [13].

There is nothing inherently wrong in adopting either of these approaches for distinguishing models of aftercare. But given that there is insufficient evidence around which models are the most appropriate, [2, 5] the lack of a clear and consistent method of categorizing models of aftercare risks defusing the evidence that is available. A standardized method of categorization would allow for more accurate description of programs. It would make explicit the defining aspect of similar categorizations used in previous studies, e.g. primary care physician-led, primary care follow-up, community-based, and community referral model, and which are all likely referring to the same or very similar types of programs. In order for progress to be made in the evaluation of models of aftercare, there needs to be a standardized way for classifying and describing various models of aftercare for survivors of childhood cancer, particularly across studies.

### A New Taxonomy

There are relatively few examples of classifications of models of care having been developed even in other disease areas. Those that have been reported on in the academic literature, e.g., relating to maternal care or community-based mental health services, have used multi-year approaches to engage a range of stakeholders on how models should be defined and to define the data elements to capture in administrative systems [14, 15]. These studies were done in the context of reporting data to government agencies related to evaluations of outcomes and payments for services. We developed our proposed taxonomy in the context of planning a systematic review of models of aftercare. In developing our classification, we however ran into the same issue as faced by others. In particular, the need to balance the development of a system of classification that can identify features that can meaningfully group programs together, while capturing the “level of granularity” about programs required to conduct an appropriate evaluation between them [14].

In developing our taxonomy, we first reviewed previous categorizations and identified key program features they included. Based on this review, we identified six fundamental features: 1) the provider primarily responsible for managing aftercare; 2) the other providers who are regularly involved in providing aftercare; 3) the location of care; 4) the method of engaging survivors, including how survivors receive aftercare and how a program tracks its survivorship population; 5) the aftercare services provided; and 6) who receives care through the aftercare program, e.g., whether a program is risk stratified or focused only on a select group of survivors. There are clearly other relevant program characteristics that impact the care survivors of childhood cancer receive, including age restrictions on follow-up; frequency of follow-up; available resources for the program; whether transition occurs within the same institution, e.g., between the pediatric and adult oncology programs within the same cancer hospital, or to a different institution; and whether research and evaluation are part of the program. In not including these features in our framework, we do not mean to imply that they are not important or that they cannot also be captured depending on the study aim. Rather we hope to develop a framework for categorizing models of aftercare that can provide a consistent way of characterizing different types of aftercare programs without being too restrictive. For example, one of our concerns with the definition of comprehensive survivorship programs is that is too detailed in its criteria to include anything other than these specific programs [12].

The first feature of our categorizations is the specialization of the provider who is primarily responsible for providing and organizing aftercare. Aftercare is initially provided by the pediatric team, but after adolescence, cancer programs differ in terms of who is responsible for providing care. Eshelman-Kent et al. report that 35% of survivors continue to be followed into adulthood by the pediatric care team [4]. If survivors are followed by a pediatrician or a pediatric long-term care clinic into adulthood, we would classify this model as a pediatric-led model of aftercare. For models in which the survivor transfers to a new care team, it may be an adult oncologist, a primary care physician, or a nurse who is the provider primarily responsible for overseeing the survivor’s aftercare. Another common arrangement is a hybrid where a primary care physician follows the survivor for their survivorship care, but this physician maintains a close connection to an oncology program that can be called upon if any serious issues arise [1]. For models of care which follow survivors only by telephone, mail or e-mail, we would classify these models as distant follow-up. If there is no regular follow-up with survivors, we would classify these models as minimal follow-up.

Table 2 shows our proposed categorization. There are a number of reasons for starting with the provider as the primary basis for classifying different models of aftercare. First, it is a basis for distinguishing models of aftercare used, or partly used, by other authors [2, 5, 11]. It also incorporates the main basis used for distinguishing models of aftercare. For example, those who classify models of aftercare in terms of the setting where the care is given are likely really concerned with whether the survivor is receiving follow-up specialist care versus primary care. Furthermore, identifying the provider makes it more specific the level of specialization the provider has, e.g., whether they are a nurse, a pediatric oncologist or adult oncologist, if survivors are being followed in the hospital setting. In most situations, it should not be too difficult to determine who is the main provider responsible for aftercare within a particular program. Even for those survivors seen in a survivorship clinic, researchers and program directors should be able to identify the person who is ultimately responsible for coordinating care in most cases.

**Table 2:** Proposed Categories of Models of Aftercare

			Models of Care
Models of Aftercare	1. Which provider is primarily responsible for aftercare?	a) Pediatrician b) Adult Oncologist c) Primary Care Physician d) Primary Care Physician and Oncologist e) Nurse f) Phone/Text/E-mail g) None	a) Pediatric-Led Model b) Adult Oncology-Led Model c) Primary Care-Led Model d) Hybrid Oncology/Primary Care Model e) Nurse-Led Model f) Distant Follow-up Model g) Minimal Follow-up Model
Other Key Features	2. Which providers are regularly involved in providing aftercare?		
	3. The location of care		
	4. How are survivors engaged?		
	5. Which services are provided?		
	6. Who receives services?		

While identifying the lead provider as the basis for classifying models of care, as stated above, there are other relevant features that are useful to include in identifying models of aftercare. Yet rather than developing specific categorizes for each of these features, in practice we found that this approach led to many difficult choices in categorizing programs. Instead of forcing these artificial distinctions on programs, and thereby missing much of the relevant information, for the other five features we simply capture the detail information around each. Our proposed approach then is to classify programs broadly in terms of their lead provider, then identifying other key aspects where details need to be captured. It is hoped that this approach will allow for some groupings of relevant programs, while allowing for the appropriate level of detail to be able to distinguish key program features.

## Discussion

In developing our approach to categorizing the different models of aftercare, we have tried to incorporate features that other authors have seen as important. For this reason, it should be able to be applied to categorize programs reviewed in previous studies even if they used a different basis of categorization. We have also tried to format the approach using questions that can be clearly answered, so that this approach should be fairly straight forward to use. While it is possible that questions may arise whether a program is multidiscipline or who is the lead provider, in practice program characteristics around these key categorizes should be identifiable for most programs.

Still, given the variation in programs that are possible, it is likely that questions of proper categorization will arise. For example, if you had an aftercare program in a pediatric hospital the lead physician who is a primary physician by training, under our classification this would be primary led, even though the provider would have a good deal of specialty knowledge. If the lead physician were to change, this may result in a reclassification of the entire model. Those reporting on different models of care should be cognisant of these factors and report on them when they do occur.

We developed this categorization based on the articles we identified in a systematic review on models of aftercare for survivors of childhood cancer. It is possible that we have not identified in this review all the potential models of aftercare that have been proposed in the academic literature. We also recognize that the Distant Follow-up Model could be seen as a mode of communicating with patients, rather than a type of provider. We have included this model as a type of provider because it is different than regularly meeting with a nurse or other provider, even though there is likely a health care provider who is reviewing these responses. The basis for the categorization we propose is what seems most reasonable to us given the work that has been previously published on models of aftercare and the way that others have categorized them. Our goal is to highlight the need for a consistent categorization in this area and to propose an approach for doing this. We recognize that other approaches are possible and would welcome further discussion around ways to improve our proposed categorization and to maximize the utility of the research being conducted on this topic.

Survivors of childhood cancer face a number of unique health risks that make their required aftercare unique from other patient groups, including adult cancer survivors. We know that poorer health outcomes are associated with unmet needs of childhood cancer survivors and families during the period when they need aftercare [3]. Optimal participation and structure of models of aftercare offers childhood cancer survivors an opportunity to enhance their health care and ultimately reduce their risks of late effects associated with their cancer treatment. Yet issues around transition and follow-up care occur for many groups of patients, include most pediatric patients with chronic conditions who require ongoing follow-up. The model that we propose here for categorizing models of care for survivors of childhood cancer could be modified to be applied to other patients groups. For example, for patients with type 1 diabetes similar issues arise regarding the specialization of the provider who is responsible for managing their condition, and around continued access to multidisciplinary care [16]. How structured programs are and whether they stratify patients based on their level of risk or acuity are also questions faced in other areas. Research in these areas should explore the possible adoption of a method of categorization similar to the approach we are proposing for survivors of childhood cancer.

## Conclusions

There is a good deal of interest in the effectiveness of different models of aftercare. Future research in this area would be assisted by the adoption of a shared taxonomy that will allow programs to be identified by their structural type [5]. Because of existing staff, resources, and geographic location, there may be little flexibility regarding the adoption of new models of aftercare. Regardless, programs could likely implement interventions which have been evaluated in programs with similar structures. Reviews, like Singer et al., maybe a step in this direction [17]. Yet at the very least the adoption of a standardized method for classifying different models of aftercare will help avoid unnecessary confusion and help ensure the maximum utility of the evaluations that are conducted.

## Data Availability

Data sharing is not applicable to this article as no datasets were generated or analysed during the current study.
